# p35 Regulates the CRM1-Dependent Nucleocytoplasmic Shuttling of Nuclear Hormone Receptor Coregulator-Interacting Factor 1 (NIF-1)

**DOI:** 10.1371/journal.pone.0110584

**Published:** 2014-10-16

**Authors:** Xiao-Su Zhao, Wing-Yu Fu, Winnie W. Y. Chien, Zhen Li, Amy K. Y. Fu, Nancy Y. Ip

**Affiliations:** 1 Division of Life Science, The Hong Kong University of Science and Technology, Clear Water Bay, Hong Kong, China; 2 Molecular Neuroscience Center, The Hong Kong University of Science and Technology, Clear Water Bay, Hong Kong, China; 3 State Key Laboratory of Molecular Neuroscience, The Hong Kong University of Science and Technology, Clear Water Bay, Hong Kong, China; McGill University Department of Neurology and Neurosurgery, Canada

## Abstract

Cyclin-dependent kinase 5 (Cdk5) is a proline-directed serine/threonine kinase, which plays critical roles in a wide spectrum of neuronal functions including neuronal survival, neurite outgrowth, and synapse development and plasticity. Cdk5 activity is controlled by its specific activators: p35 or p39. While knockout studies reveal that Cdk5/p35 is critical for neuronal migration during early brain development, functions of Cdk5/p35 have been unraveled through the identification of the interacting proteins of p35, most of which are Cdk5/p35 substrates. However, it remains unclear whether p35 can regulate neuronal functions independent of Cdk5 activity. Here, we report that a nuclear protein, nuclear hormone receptor coregulator (NRC)-interacting factor 1 (NIF-1), is a new interacting partner of p35. Interestingly, p35 regulates the functions of NIF-1 independent of Cdk5 activity. NIF-1 was initially discovered as a transcriptional regulator that enhances the transcriptional activity of nuclear hormone receptors. Our results show that p35 interacts with NIF-1 and regulates its nucleocytoplasmic trafficking via the nuclear export pathway. Furthermore, we identified a nuclear export signal on p35; mutation of this site or blockade of the CRM1/exportin-dependent nuclear export pathway resulted in the nuclear accumulation of p35. Intriguingly, blocking the nuclear export of p35 attenuated the nuclear accumulation of NIF-1. These findings reveal a new p35-dependent mechanism in transcriptional regulation that involves the nucleocytoplasmic shuttling of transcription regulators.

## Introduction

Neural development in response to various stimuli such as neural activity, neurotrophic factors, and nuclear hormones is a tightly coordinated process that involves the concerted regulation of gene expression [1], [2]. One major regulatory pathway that governs gene transcription is the nuclear accessibility of transcriptional factors or regulators such as histone-modifying enzymes. The nucleocytoplasmic shuttling of negative transcriptional regulator class II histone deacetylases in response to neural activity is important for the regulation of gene expression during neuronal differentiation and synaptogenesis [3]. However, the precise mechanisms regulating the nuclear accessibility of transcriptional complexes in neurons, including post-translational modifications such as protein phosphorylation/dephosphorylation and protein–protein interactions, remain largely unknown.

Cyclin-dependent kinase 5 (Cdk5) is a proline-directed serine/threonine kinase. It has two neuronal-specific Cdk5 activators, p35 and p39 [4], whose associations with Cdk5 are essential for the activity of the kinase. Moreover, p35 is highly expressed in the brain at the late embryonic and early postnatal stages, which is the period critical for neuronal cell positioning [5], [6]. Furthermore, p35 knockout mice die by adulthood and exhibit neuronal migration defects, suggesting that p35 is important for early neural development [7]. The functions of Cdk5/p35 have been uncovered through the identification of the substrates of Cdk5, most of which were first identified to interact with p35 [8].

The p35-associated Cdk5 activity is mainly localized to membrane fractions. Nonetheless, Cdk5 and p35 are also expressed in the nuclei of neurons [9], [10]. Interestingly, the nuclear localization of Cdk5/p35 has been suggested to be important for regulating various activities of transcription factors and chromatin remodeling [11], [12], [13], [14], [15], [16], [17]. Thus, understanding the regulatory pathways that control the nucleocytoplasmic trafficking of Cdk5 and/or p35 may provide insights to their functional roles in neural development. It is noteworthy that the nuclear import of p35 is mediated by the importin pathway and that endogenous p35 is shuttled between the nucleus and cytoplasm upon growth factor stimulation [9], [18]. While the mechanism that regulates the nuclear export of p35 has not been investigated, the major regulatory mechanism of nuclear–cytoplasmic protein transportation is mediated by the nuclear export receptor, chromosome region maintenance 1 (CRM1) protein [19]. CRM1 binds its cargo protein through recognition of a hydrophobic nuclear export signal (NES) peptide sequence [20].

The present study aimed to discover novel roles of p35 in neural development through identification of its new interacting partner(s). We identified a new p35-interacting protein, nuclear hormone receptor coregulator (NRC)-interacting factor 1 (NIF-1). These findings reveal an unexpected role of p35 in mediating the nucleocytoplasmic shuttling of NIF-1. NIF-1 protein, which was originally identified to associate with NRC [21], is prominently expressed in the nuclear fractions of early differentiating neurons [21] and involved in neurogenesis [22]. The results show that p35 regulates the subcellular localization of NIF-1. Overexpression of p35 stimulates the nuclear export of NIF-1 via a CRM1-dependent pathway, and this nuclear/cytoplasmic shuttling of NIF-1 is mediated by the NES identified on p35. Importantly, inhibition of the CRM1-dependent pathway triggers the nuclear accumulation of p35 in neurons. These findings collectively reveal a previously unidentified role of p35 in the regulation of the nucleocytoplasmic trafficking of proteins and provide insights into how p35 regulates gene transcription.

## Materials and Methods

### siRNAs and DNA constructs

Complementary DNAs (cDNAs) encoding p35, p25, and p10 were subcloned into the yeast GAL4 DNA-binding vector, pAS2-1 (Clontech, Palo Alto, CA, USA). Full-length p35 was used as bait in the yeast two-hybrid screen [23]_ENREF_34. Partial cDNA fragments of NIF-1 encoding different C-terminal regions of NIF-1 were amplified by PCR and subcloned into the GAL4 transcriptional activation vector, pACT2 (Clontech). Full-length NIF-1 was amplified by reverse transcription-PCR and subcloned into the expression vector, pEGFP-C3. The GST-NIF-1-C/His-NIF-1-C (amino acids 772–1,291), GST-NIF-1-N (amino acids 1–772), and GST-NIF-1A (amino acids 1,066–1,291) fusion constructs were generated by subcloning the corresponding NIF-1 fragments into pGEX-6p-1 and pETH-32, respectively (GE Healthcare). The NES mutant construct of p35 was generated by PCR using complementary primers containing the mutations (5′-TCCAGGCGGTCGCAGCAACCTGCGCATACCTCTCCTACT, 3′-AGTAGGAGAGGTATGCGCAGGTTGCTGCGACCGCCTGGA), and the cDNA fragment was subcloned into pEGFP-N3. All constructs were confirmed by sequencing.

Antibodies specific for Cdk5 (i.e., C-8 and DC-17), p35 (i.e., C-19), and HA were purchased from Santa Cruz Biotechnology (Santa Cruz, CA, USA). A polyclonal antibody against p39 was raised against a synthetic peptide (KGRRPGGLPEE, amino acids 14–24). A custom antibody against NIF-1 was raised against the GST fusion protein of NIF-1 C-terminus (amino acids 1,066–1,291). The specificity of the antibody was confirmed by pre-absorption with the His fusion protein of the NIF-1 C-terminus (amino acids 772–1,291).

### Yeast two-hybrid screen

A yeast two-hybrid screen was performed following the Matchmaker two-hybrid screen protocol (Clontech). p35 was used as bait to screen for a mouse muscle cDNA library in a GAL4 transcriptional activation vector, pACT2. The bait and library plasmid were co-transformed into yeast strain Y190. The transformants were selected on SD-Trp-Leu-His plates, and the selected clones were subjected to a β-galactosidase filter assay. The C-terminal region of NIF-1 encoding amino acids 772–1,291 was isolated from the two-hybrid screen. To map the interacting domains between p35 and NIF-1, cDNA fragments corresponding to different domains of p35 and NIF-1 were subcloned into the yeast DNA binding-domain vector, pAS2-1, and transcriptional activation-domain vector, pACT2, respectively. Subsequent two-hybrid interaction analyses were carried out by co-transformation of plasmids containing the GAL4 DNA-binding (i.e., pAS2-1) and activation (i.e., pACT2) domains into yeast.

### Cell culture and transfection

COS-7 and Neuro-2a cells (ATCC, Manassas, VA, USA) were cultured in DMEM (Invitrogen) supplemented with 10% heat-inactivated FBS plus antibiotics in an incubator at 37°C with 5% CO_2_. Primary cortical neuron cultures were prepared from embryonic day 18 (E18) SD rats. E18 timed pregnant rats were euthanized by carbon dioxide inhalation and rapid decapitation. The E18 embryos subsequently removed and sacrificed by decapitation. Cortices were then dissociated in trypsin and plated on poly-d-lysine–coated culture plates. Cells were cultured in Neurobasal medium supplemented with 2% B27 and antibiotics. All animal experiments were conducted in accordance with the protocol number 2009056 approved by the Animal Care Committee of the Hong Kong University of Science and Technology. To examine the effects of blocking the nuclear export and inhibition of Cdk5 activity, the cells were treated with leptomycin B (LMB, 5–10 ng/mL; Sigma, St. Louis, MO, USA) or roscovitine (10–25 µM; Sigma), respectively, for 4 or 8 h before analysis.

COS-7 and Neuro-2a cells were transfected with Lipofectamine Plus reagents (Invitrogen). Meanwhile, cortical neurons were transfected with the nucleofector transfection kit (Amaxa) or a calcium phosphate transfection assay.

### Pull-down assay, protein extraction, immunoprecipitation, and western blot analysis

For the pull-down analysis, recombinant proteins encoding GST-NIF-1-C, GST-NIF-1-N, and GST-NIF-1A were expressed in *Escherichia coli* (BL21 strain) and purified using a glutathione-Sepharose 4B column according to the manufacturer's instructions (GE Healthcare). Purified protein (1–2 µg) was bound to the glutathione-Sepharose beads and subsequently incubated with (1–2 µg) recombinant p35 protein (Abcam) or 500 µg lysates from p35-expressing COS-7 cells. The beads were washed with lysis buffer, and the bound proteins were resuspended in SDS loading buffer, resolved by SDS-PAGE, and analyzed by western blot analysis.

### *In vitro* phosphorylation assay

To determine if Cdk5 can phosphorylate the C-terminal of NIF-1, 0.5 µg GST-NIF-1-C recombinant protein was used as substrate for reconstituted Cdk5/p25 in the *in vitro* phosphorylation assay. The kinase assay was performed in kinase buffer (20 mM MOPS [pH 7.4], 15 mM MgCl_2_, and 100 µM ATP) containing 1 µCi [γ-^32^P] ATP for 30 min at 30°C. GST protein and histone H1 served as negative and positive controls, respectively. The phosphorylated proteins were separated by SDS-PAGE and visualized by autoradiography.

### Immunocytochemical analysis

To determine the subcellular localizations of NIF-1, p35, p25, and p39, COS-7 cells were washed with 1× PBS with 900 µM Ca^2+^ and 500 µM Mg^2+^. The cells were fixed by 4% paraformaldehyde and 5% sucrose in PBS with Ca^2+^ and Mg^2+^ for 30 min and subsequently washed 3 times with 1× PBS. After blocking with 1% bovine serum albumin, 4% goat serum, and 0.1% Triton X-100 (Sigma) for 20 min, the cells were incubated with the corresponding primary antibody overnight at 4°C and subsequently washed 3 times with 1× PBS. Alexa Fluor secondary antibody was added with 1% bovine serum albumin for 1 h at room temperature in the dark. The cells were washed 5 times with 1× PBS, stained with DAPI, and mounted with MOWIOL for fluorescent microscopy.

### Statistical analysis

All data are expressed as the arithmetic mean ± standard error of the mean (SEM). Comparisons between groups were made using Student's *t-*test and one-way ANOVA.

## Results

### NIF-1 interacts with p35

**Figure 1:**
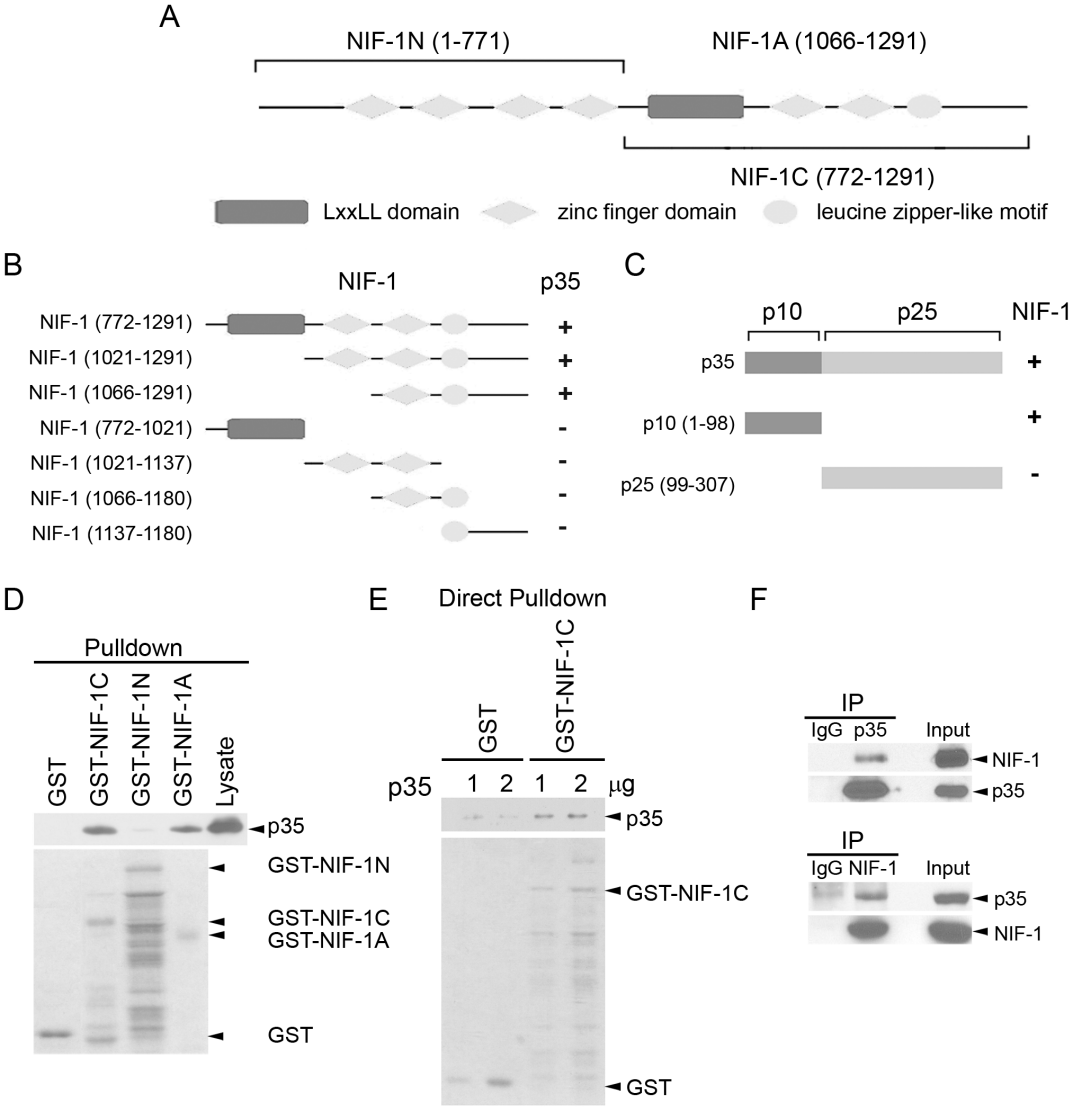
p35 interacts with NIF-1. (A) Mouse NIF-1 encodes a 1,291-amino acid protein and contains 6 zinc finger domains, an LXXLL domain, and a leucine zipper-like motif. (B & C) Mapping of interaction domains between NIF-1 and p35. Yeast was co-transformed with different domains of p35 and NIF-1. +, strong interaction; −, absence of interaction. (B) The C-terminal region of NIF-1 (amino acids 1,066–1,291) containing the 6^th^ zinc finger domain, leucine zipper-like motif, and short C-terminus was sufficient to interact with p35. (C) The N-terminal region of p35 (corresponding to the p10 fragment) was required for the interaction between p35 and NIF-1. p10 comprises the 98 N-terminal amino acids of p35, while p25 contains the C-terminal region of p35. (D & E) Direct interaction between NIF-1 and p35. Recombinant GST fusion proteins encoding different regions of NIF-1 were incubated with lysate prepared from p35-overexpressing COS-7 cells (D) or recombinant p35 protein (E). The bound proteins were pulled down by glutathione-Sepharose and analyzed by western blot analysis (Lysate, as an input control). Bottom panel: Coomassie-stained gel. (F) Association of p35 with NIF-1 in mammalian cells. COS-7 cells were transiently transfected with NIF-1 and p35. Cell lysate was immunoprecipitated (IP) with p35 or NIF-1 antibody as indicated and subjected to western blot analysis. Rabbit normal IgG (IgG) was used as a negative control.

As a first step to identify p35-interacting protein(s), full-length p35 was used as a bait to screen a postnatal day 14 mouse muscle cDNA library using a yeast two-hybrid screen [15]. The results revealed a positive clone encoding NIF-1 interacting with p35. Mouse NIF-1 encodes a 1,291-amino acid protein that contains 6 Cys-2/His-2 zinc fingers, an N-terminal stretch of acidic amino acids, and a C-terminal leucine zipper-like motif (Fig. 1A). The NIF-1 fragment that interacts with p35 spans the C-terminal half region of NIF-1 (amino acids 772–1,291; Fig. 1A & B). We further characterized the interaction between NIF-1 and p35 by expressing different fragments of them in yeast. The region of NIF-1 flanking the 6^th^ zinc finger and leucine zipper-like motif (amino acids 1,066–1,291) were sufficient for binding to p35 (Fig. 1B). On the other hand, the N-terminal region of p35, corresponding to the p10 fragment, is responsible for its interaction with NIF-1 (Fig. 1C).

To confirm that NIF-1 directly binds to p35, lysate of p35-expressing COS-7 cells or recombinant p35 protein was incubated with GST fusion proteins encoding different regions of NIF-1. Intriguingly, p35 was pulled down efficiently by the C-terminal fragments of NIF-1 (GST-NIF-1C and GST-NIF-1A; Fig. 1A, D & E), confirming that NIF-1 binds directly to p35 via its C-terminal region. Furthermore, co-immunoprecipitation analysis demonstrated that NIF-1 interacts with p35 in mammalian cells (Fig. 1F). As p39 was not co-immunoprecipitated with NIF-1 when co-expressed in COS-7 cells, the interaction between NIF-1 and p35 is specific (data not shown).

### p35 regulates NIF-1 subcellular localization

**Figure 2:**
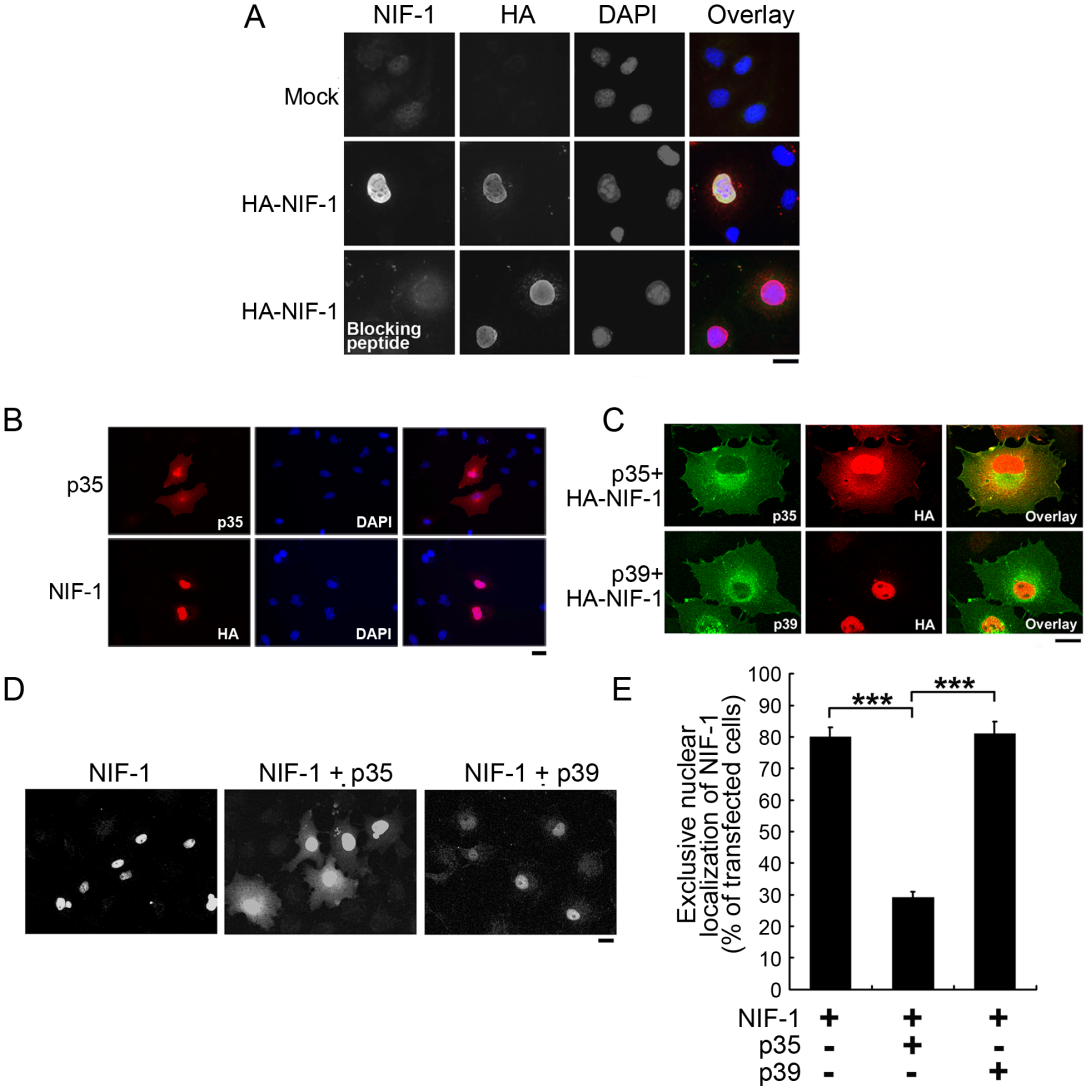
p35 regulates NIF-1 subcellular localization. (A) Nuclear localization of NIF-1 in COS-7 cells. HA-tagged NIF-1 expressing COS-7 cells were stained with HA and NIF-1 antibody. The nuclei were stained with DAPI. The specificity of the NIF-1 antibody was confirmed by pre-absorption of the antibody with the immunogen (HIS-tagged NIF-1 fusion protein; bottom panels). (B) Cellular distribution of p35 and NIF-1 in COS-7 cells. p35 or HA-tagged NIF-1 expressing COS-7 cells were stained with HA and p35 antibodies, respectively. The specificity of p35 and NIF-1 staining was confirmed by the negative staining signals of the neighboring non-transfected cells (C) The expression of p35 but not p39 abolished the exclusive nuclear accumulation of NIF-1. HA-tagged NIF-1 was co-expressed with p35 or p39 in COS-7 cells. NIF-1 was stained with HA antibody, and p35 and p39 were stained with their corresponding antibodies. (D) COS-7 cells were transfected as described in B and stained with NIF-1 antibody followed by Alexa Fluor 488-conjugated anti-rabbit IgG. Micrographs are representative images of transfected cells. (E) Quantitative analysis of the cells that exhibited exclusive nuclear accumulation of NIF-1. Cells (*n* = 100) were scored for each condition. Results represent the mean ± SEM of 3 replicates (***p <0.05, significantly different from that of the cells expressing NIF-1 alone, one-way ANOVA followed by the Student–Newman–Keuls test). Scale bar = 10 µm.

The dynamic nucleocytoplasmic shuttling of nuclear receptor coregulators is important for controlling nuclear receptor-dependent transcriptional activity, which in turn serves as a regulatory mechanism for controlling nuclear receptor functions [24]. Thus, we examined whether p35 regulates the nucleocytoplasmic shuttling of NIF-1. NIF-1 was localized exclusively to the nuclei of COS-7 cells (Fig. 2A & B), consistent with a previous report [21]. The signal was completely blocked when the NIF-1 antibody was pre-absorbed with its immunogen, indicating the nuclear staining of NIF-1 was specific (Fig. 2A, lower panels). When overexpressed in COS-7 cells, p35 was widely distributed in the cytoplasm and nucleus (Fig. 2B). Intriguingly, co-transfection of p35 and NIF-1 triggered the subcellular redistribution of NIF-1 protein in COS-7 cells. Moreover, p35 increased NIF-1 protein expression in the cytoplasm and at the membrane ruffles (Fig. 2C), suggesting that it abolished the restricted nuclear localization of NIF-1. However, the nucleocytoplasmic distribution of p35 was unaffected (Fig. 2B & C). Interestingly, p39, which does not interact with NIF-1, did not cause such redistribution of NIF-1 to the cytoplasm (Fig. 2C). The p35-dependent redistribution of NIF-1 to the cytoplasm was further confirmed by staining with NIF-1 antibody (Fig. 2D & E). Approximately 80% of NIF-1–expressing cells exhibited exclusive nuclear localization of NIF-1 (Fig. 2D & E). In contrast, the co-expression of p35 and NIF-1 reduced the percentage of cells that exhibited restrictive nuclear expression of NIF-1 to ∼30% (Fig. 2D & E). Similar redistribution of NIF-1 to the cytoplasm was observed upon co-transfection with GFP-tagged NIF-1 and p35 (data not shown). Hence, these findings suggest that p35 regulates the nucleocytoplasmic shuttling of NIF-1.

### p35 mediates NIF-1 nuclear export via a CRM-1/exportin pathway

**Figure 3:**
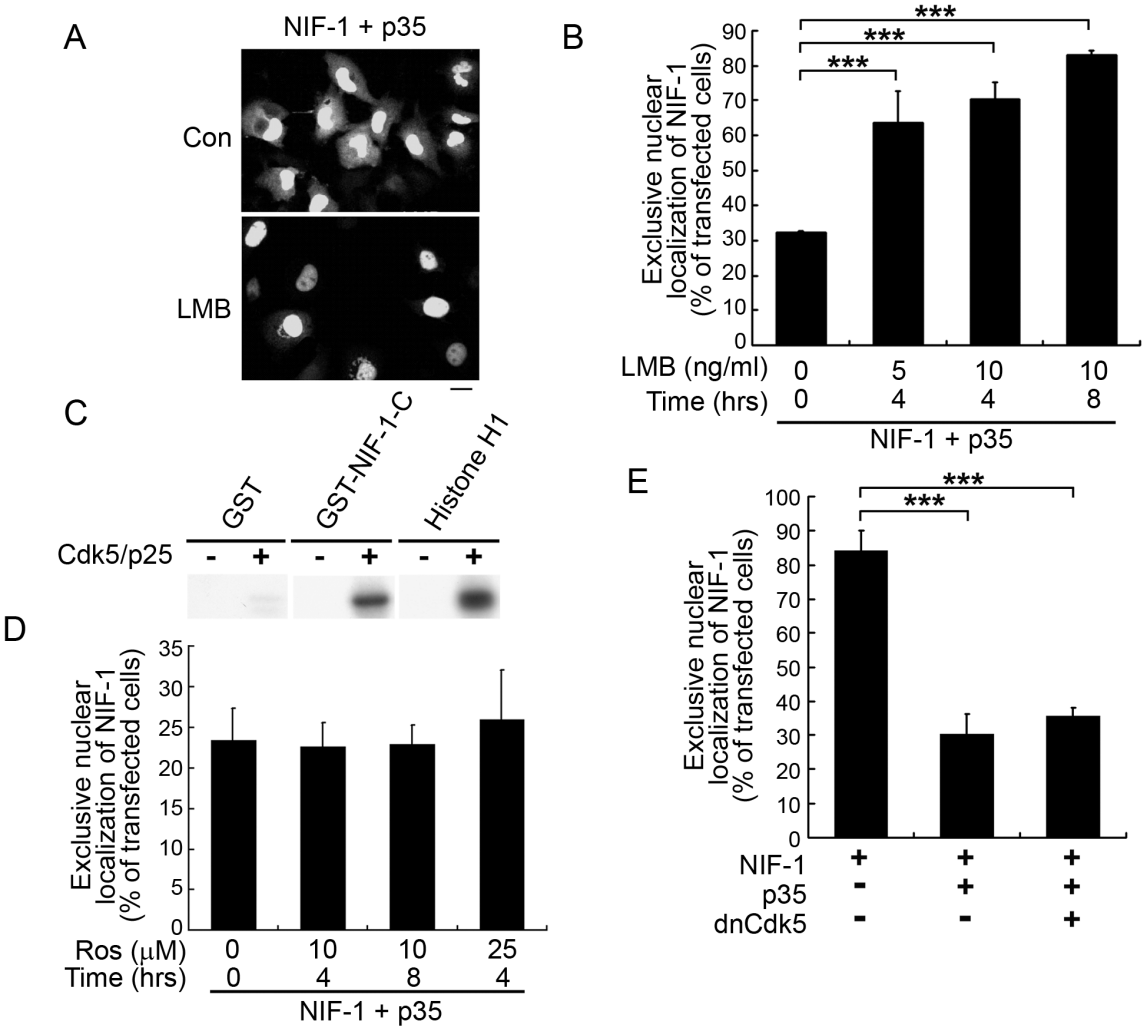
Mediation of p35-triggered NIF-1 nuclear export via a CRM1-dependent pathway. (A & B) COS-7 cells were transfected with p35 and HA-tagged NIF-1. Twenty-four hours after transfection, the cells were treated with LMB (5 or 10 ng/mL) for 4–8 h, stained with HA antibody, and examined by fluorescence microscopy. Representative fluorescence images (A) and quantitative analysis of the cells showing exclusive nuclear accumulation of NIF-1 (B). At least 100 cells were scored for each condition in each trial. Results represent the mean ± SEM of 3 replicates (***p <0.05, one-way ANOVA followed by the Student–Newman–Keuls test). (C–E) p35-stimulated redistribution of NIF-1 from the nucleus to cytoplasm is independent of Cdk5 activity. (C) Active Cdk5 phosphorylated recombinant NIF-1 protein. Recombinant GST-NIF-1-C protein was subjected to phosphorylation assay by Cdk5 (GST and histone H1 were used as negative and positive controls, respectively). (D) COS-7 cells were transfected with the HA-tagged NIF-1 and p35, and subsequently treated with roscovitine (Ros, 10 or 25 µM) for 4 or 8 h. The cells with exclusive nuclear accumulation of NIF-1 were quantified. (E) Inhibition of Cdk5 activity by dnCdk5 expression did not affect the p35-stimulated nuclear export of NIF-1. COS-7 cells were transfected with NIF-1, p35, and dnCdk5 constructs as indicated. Quantitative analysis as described in (D). Results represent the mean ± SEM of 3 replicates (***p <0.05, one-way ANOVA followed by the Student–Newman–Keuls test).

As p35 promoted the redistribution of NIF-1 from the nucleus to the cytoplasm in COS-7 cells, we examined whether p35 triggers NIF-1 nuclear export. The CRM1-dependent nuclear export pathway is a major pathway for transporting proteins out of the nucleus [25]. Treatment with LMB, a specific inhibitor of CRM1-mediated export [26], abolished the p35-dependent export of NIF-1 from the nucleus to the cytoplasm, resulting in the nuclear accumulation of NIF-1 (Fig. 3A & B). LMB treatment increased the restricted nuclear localization of NIF-1 in COS-7 cells expressing both NIF-1 and p35 (from ∼30% to>60%) similar to that observed in the cells expressing NIF-1 alone (Fig. 2D & 3B). These findings suggest that p35 triggers NIF-1 nuclear export via a CRM1-dependent nuclear export pathway.

### The NES of p35 is responsible for NIF-1 nuclear export

**Figure 4:**
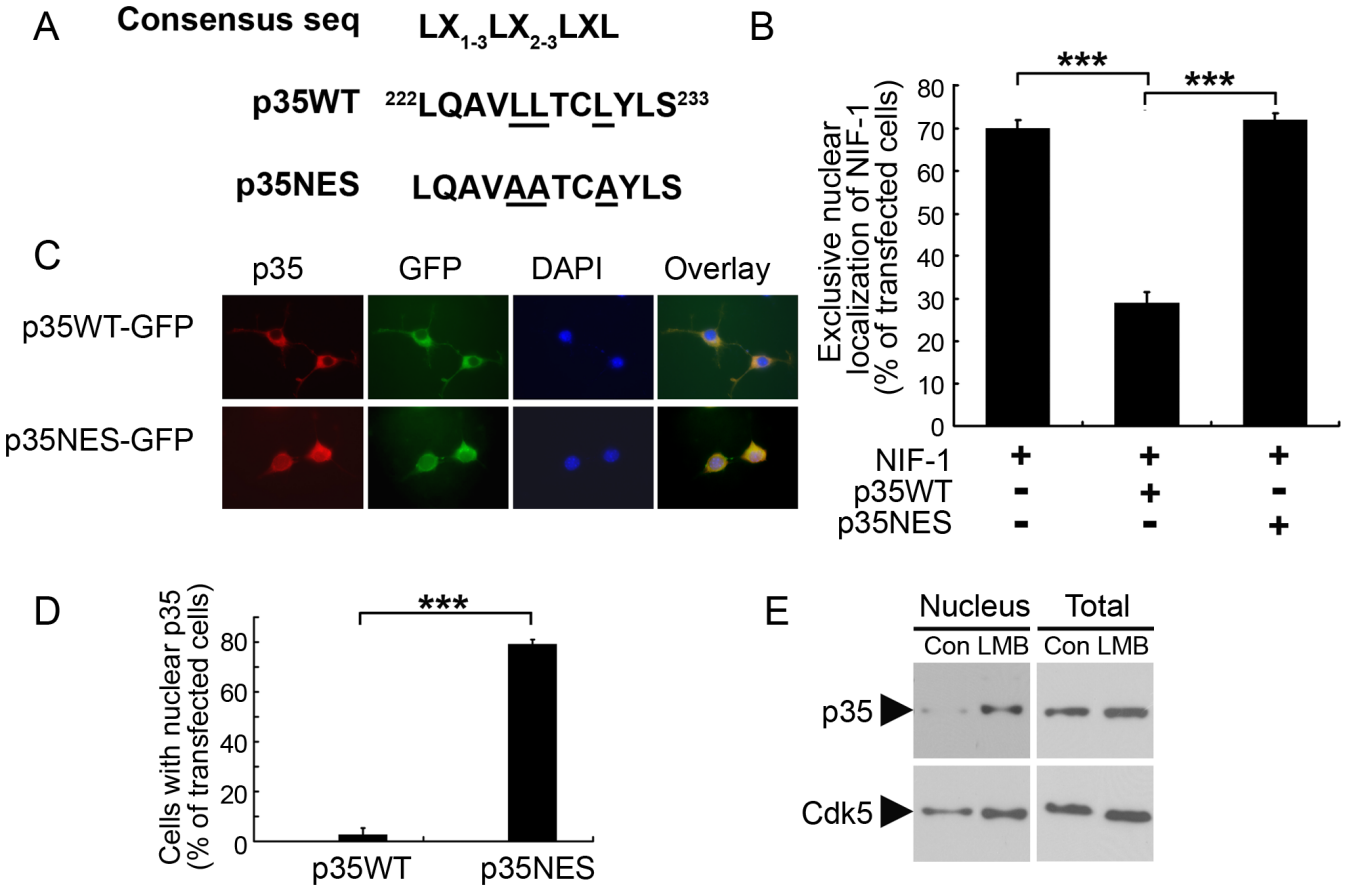
Dependence of p35 nuclear export on its NES. (A) Consensus sequence of the NES on p35. The functional NES comprises a core of closely spaced leucine residues or other hydrophobic amino acids. The critical hydrophobic residues in the putative NES of p35 are underlined. An NES mutant of p35, p35NES, was generated by mutating the 3 conserved hydrophobic residues (i.e., leucine 226, 227, and 230) to alanine. (B) The NES of p35 is required to mediate the nuclear export of NIF-1. COS-7 cells were co-transfected with HA-NIF-1 and p35WT or p35NES and subsequently stained with HA antibody. The results of the quantitative analysis represent the mean ± SEM of 3 replicates (***p <0.05, one-way ANOVA followed by the Student–Newman–Keuls test). (C) Mutating the NES of p35 increased the population of Neuro-2A cells containing nuclear p35. Neuro-2A cells were transfected with p35WT-GFP or p35NES-GFP and subsequently differentiated by RA. The cells were stained with p35 antibody. Representative fluorescent images depicting the localization of p35WT and p35NES (indicated by the p35 staining and GFP expression). (D) Quantitative analysis of cells with nuclear p35. Cells with nuclear p35 were counted if the GFP signal in the nucleus was>75% than that of the cytoplasm. Results represent the mean ± SEM of 3 replicates (***p <0.05, Student's *t*-test). (E) LMB treatment caused the accumulation of p35 in the nucleus of cortical neurons. Cultured cortical neurons were treated with LMB for 1 h, and subcellular fractionation was performed. Western blot analysis of p35 and Cdk5. Total: protein extracted from the same batch of neurons using RIPA.

As p35 is a major activator of Cdk5, we determined if NIF-1 nuclear export by p35 is dependent upon Cdk5 activity. Because of the endogenous Cdk5 expression in COS-7 cells, the transfection of Cdk5 activators alone increased Cdk5 activity (data not shown). Although active Cdk5/p25 phosphorylated NIF-1 (Fig. 3C), the inhibition of Cdk5 by roscovitine or co-expression of its dominant-negative mutant dnCdk5 failed to abolish p35-dependent NIF-1 nuclear export (Fig. 3D & 3E). These findings collectively suggest that the nuclear export of NIF-1 by p35 is regulated independent of Cdk5 activity and is instead mediated by a CRM1 exportin-dependent pathway. CRM1 functions as a receptor for leucine-rich NESs. The NES of p35 is characterized by the presence of four critically spaced large-group hydrophobic residues most often represented by leucine (Fig. 4A) [27]. Interestingly, no potential NES was found in mouse NIF, whereas a typical leucine-type NES was identified at amino acid residues 222–233 of p35 (Fig. 4A). Therefore, we generated a p35 NES mutant in which the three leucine residues of the NES were mutated to alanine (p35NES mut; Fig. 4A). Interestingly, co-expression of the p35 NES mutant failed to trigger NIF-1 export to the cytoplasm (Fig. 4B), suggesting that the redistribution of NIF-1 to the cytoplasm is dependent on the nuclear export property of p35.

Next, we examined whether p35 indeed shuttles between the nucleus and cytoplasm in neuronal cells. While p35WT was prominently detected in the cell bodies and along the neurites of Neuro-2a cells, its expression was barely detected in the nucleus. In contrast, p35NES was prominently expressed in the nuclei of Neuro-2a cells (Fig. 4C & D). More importantly, blocking the CRM1-dependent nuclear export pathway by LMB resulted in significant accumulation of p35 in the nuclei of neurons while the total expression of p35 remained relatively unchanged (Fig. 4E). These findings collectively suggest that the CRM1-dependent pathway is responsible for the nuclear export of p35 in neurons, which may in turn regulate the subcellular localization and nuclear functions of NIF-1.

## Discussion

The neuronal activator of Cdk5, p35, is believed to exert its functions through its interaction with Cdk5, resulting in the phosphorylation of various cellular substrates [28], [29]. In the nucleus, Cdk5/p35 controls transcriptional regulation at multiple levels, including the phosphorylation of transcription factors and regulation of the histone acetylation through their interaction with histone deacetylase complexes [9], [14], [15], [30]. The present study identified a new mechanism of p35 wherein it regulates nuclear functions through modulating the nuclear accessibility of the transcriptional regulator NIF-1. In particular, p35 interacts with NIF-1 and regulates the subcellular localization of the protein independent of Cdk5-dependent phosphorylation. Thus, the present findings reveal a new mechanism of p35 in transcriptional regulation that does not require the kinase activity of Cdk5.

Deficiency of p35 results in aberrant brain development and adult lethality, suggesting that the protein is critical for neural development [7]. While p35 is ubiquitously expressed in neurons, its nuclear function is evidenced by the functional importance of nuclear Cdk5/p35 in the maintenance of neuronal survival [31], [32]. Although the mechanism that regulates the subcellular distribution of p35 is poorly understood, post-translational modification of p35 by myristoylation is suggested to determine the attachment of the protein to the membrane [16]. The present findings identified an NES on p35 that controls the nucleocytoplasmic shuttling of p35. Furthermore, the trafficking of p35 is believed to be responsible for mediating the nucleocytoplasmic shuttling of its interacting proteins including Cdk5 and NIF-1, thus revealing a new molecular mechanism by which p35 modulates gene transcription.

Transcriptional activation induced by neuronal activity and growth factors is a pivotal control mechanism of neuronal development [33]; this process is specifically regulated by multiple mechanisms including post-translational modifications such as phosphorylation, ubiquitination, and SUMOylation [24], [34]. Another level of regulation lies in the control of the subcellular localization of transcriptional regulator through intracellular trafficking. Indeed, our previous findings demonstrate that the growth factor neuregulin stimulates the nuclear accumulation of p35 [35]. Our unpublished data also suggest that depolarization induces the translocation of p35 and NIF-1 from the cytoplasm to the nucleus in cortical neurons. Thus, it is interesting to speculate that neuronal activity or growth factors are able to relay extracellular signals to the nucleus through the regulation of nucleocytoplasmic trafficking of p35 and thus its associated proteins (e.g., NIF-1), thereby modulating transcriptional mechanisms in neurons. The p35-mediated nucleocytoplasmic shuttling of NIF-1 is able to modulate the access of NIF-1 to the transcriptional complex and thus the regulation of gene activation and/or transcription termination. However, further studies are required to determine which signals regulate p35-mediated nuclear export.

Many transcription factors that were initially found to be localized in the nucleus were subsequently shown to be expressed in the cytoplasmic regions and exert specific functions. For example, the localization of the transcription factors Stat3 and neurogenin-3 to cytoplasmic compartments is important for their functions in tumorigenesis and synaptogenesis, respectively [36], [37]. NIF-1 plays an important role in early neurogenesis through transcriptional regulation [22]. However, whether the protein exerts cytoplasmic functions or has functional roles during the later stage of brain development remains to be elucidated. It is noteworthy that NIF-1 was prominently expressed in the nuclear fraction of embryonic rat brains and decreased upon postnatal development, whereas p35 increased gradually from the late stage of embryonic development to postnatal development [6]. Thus, it is interesting to speculate that the nuclear export of NIF-1 by p35 may lead to the termination of the nuclear functions of NIF-1 and resulting in protein degradation.

Taken together, the present findings reveal a newly identified NES on p35 that regulates the nucleocytoplasmic shuttling of p35. This protein trafficking mechanism may result in the redistribution of p35 and its interacting partners (e.g., NIF-1) between the nucleus and cytoplasm, thus demonstrating a new molecular mechanism by which p35 modulates gene transcription.
